# The 3i Conceptual Framework for Recognizing Patient Perspectives of Type 1 Diabetes During Emerging Adulthood

**DOI:** 10.1001/jamanetworkopen.2019.6944

**Published:** 2019-07-12

**Authors:** Benjamin Markowitz, Cheryl Pritlove, Geetha Mukerji, James V. Lavery, Janet A. Parsons, Andrew Advani

**Affiliations:** 1 Applied Health Research Centre, Li Ka Shing Knowledge Institute, St Michael’s Hospital, Toronto, Ontario, Canada; 2 Keenan Research Centre for Biomedical Science and Li Ka Shing Knowledge Institute of St Michael’s Hospital, Toronto, Ontario, Canada; 3 Women's College Hospital Institute for Health Systems Solutions and Virtual Care, Toronto, Ontario, Canada; 4 University of Toronto, Institute of Health Policy, Management and Evaluation, Toronto, Ontario, Canada; 5 Hubert Department of Global Health, Rollins School of Public Health and Center for Ethics, Emory University, Atlanta, Georgia

## Abstract

**Importance:**

Early emerging adulthood, the developmental life stage roughly spanning between the ages of 18 and 24 years, can be a challenging time for individuals living with type 1 diabetes, being associated with an increased risk of acute complications, loss to follow-up, and the emergence of long-term diabetes complications. Few evidence-based interventions exist, and practice guidelines instead emphasize the importance of delivering individualized, developmentally appropriate care.

**Objective:**

To inform the provision of tailored care and education by illuminating the individual lived experiences of emerging adults with type 1 diabetes.

**Design, Setting, and Participants:**

This qualitative study used in-depth interviews conducted with 33 emerging adults living with type 1 diabetes between October 14, 2016, and May 16, 2017. Participants were recruited from 2 urban Young Adult Diabetes Clinics in the city of Toronto, Ontario, Canada, using a convenience sampling approach. Narrative analysis of participants’ accounts was conducted inductively to define story typology.

**Main Outcomes and Measures:**

Participant accounts of type 1 diabetes during childhood, adolescence, and emerging adulthood.

**Results:**

Interviews were conducted with 33 participants aged 18 to 24 years (mean [SD] age at interview, 20.6 [1.7] years); 17 (51%) were women. Duration of diabetes ranged from 3 to 20 years (mean [SD] duration, 12.0 [4.8] years). Narrative analysis identified 3 distinct story types, or lenses, termed *ingrained* (n = 14), *intrusive* (n = 12), and *inconspicuous* (n = 7). Ingrained narratives portrayed an active integration of diabetes self-management into daily life. Intrusive narratives were characterized by stories of struggles for acceptance and striving for control. Inconspicuous narratives were differentiated by descriptions of a willingness to ignore diabetes and its attendant daily tasks with the goals of preserving normalcy and limiting distress. Mean levels of hemoglobin A_1c_ were higher in participants conveying an intrusive lens narrative (mean [SD], 9.3% [1.6%]) than those conveying an ingrained lens (mean [SD], 7.4% [0.7%]) (difference between intrusive and ingrained, 1.9%; 95% CI, 0.8%-2.9%; *P* = .007) or an inconspicuous lens (mean [SD], 8.6% [1.5%]).

**Conclusions and Relevance:**

Although every person is unique, there are commonalities in how emerging adults perceive and depict their experiences living with type 1 diabetes. Among the emerging adults interviewed in the present study, the 3i conceptual framework of ingrained, intrusive, and inconspicuous lenses encapsulated these commonalities. Recognition of the different psychosocial contexts surrounding type 1 diabetes during emerging adulthood may help health care professionals individualize their care accordingly.

## Key Points

**Question:**

How do individuals narrate their experiences of living with type 1 diabetes during early emerging adulthood (the developmental life stage roughly spanning between the ages of 18 and 24 years)?

**Findings:**

This qualitative study involved in-depth interviews with 33 emerging adults with type 1 diabetes. Narrative analysis identified 3 distinct story types encompassing differing perceptions of living with type 1 diabetes (3i), termed *ingrained *(characterized by actively integrating diabetes within one’s life), *intrusive *(characterized by struggles to accept diabetes and a sense of striving for control), and *inconspicuous *(characterized by attempts to minimize attention toward diabetes to protect one’s sense of normalcy).

**Meaning:**

The 3i conceptual framework provides a means by which differing emerging adult perceptions of type 1 diabetes can be recognized and articulated, which may help health care professionals better individualize their approaches to self-management support during the transitioning years.

## Teaser

This qualitative study uses narrative analysis of interviews with emerging adults, aged 18 to 24 years, with type 1 diabetes to identify distinct story types that describe participants’ perceptions of living with type 1 diabetes.

## Introduction

At its biomedical core, type 1 diabetes is a chronic disease that is characterized by insulin deficiency and elevated blood glucose. In addition to the biomedical nature of the disease, there is a human experience influenced by social interactions and relationships.^1^ Early emerging adulthood (roughly between the ages of 18 and 24 years) presents a unique set of circumstances for the type 1 diabetes experience, exemplifying the intertwining between the biomedical and the social. This period of life is characterized by emotional, social, geographical, and fiscal changes^2^ and its demands can conflict with the often inflexible demands of diabetes self-management, coinciding also with an expectation that the individual will transition from the stability of their familiar pediatric care professional to a new adult health care setting.^3^ Against such a backdrop, it is little wonder that traditional evidence-based medicine strategies have fallen short, leaving early emerging adults with type 1 diabetes at increased risk of loss to follow-up,^4^ deterioration in glycemic control,^5^ acute complications,^6^ and the emergence of long-term diabetes complications.^7^ Indeed, in the large T1D Exchange registry, only 14% of individuals aged 18 to 25 years achieved the guideline-recommended target hemoglobin A_1c_ (HbA_1c_) level of less than 7%, the lowest percentage of any age group.^8^

Despite the attendant risks of the early emerging adult years, research into models of transition care has mostly failed to yield the evidence necessary to inform firm guideline recommendations. Instead, most clinical practice guidelines for transition-age patients with type 1 diabetes emphasize that care must be “individualized and developmentally appropriate,”^9^ yet there is no standard definition for developmentally appropriate health care.^10^ To better understand the lived experiences of emerging adults with type 1 diabetes, we set out to collect and analyze the stories that they tell. Aligned with the near-ubiquitous use of social media platforms by this population, we conjectured that the self-perceptions of emerging adults are inextricably entangled with their stories (or narratives).^11,12^ Narratives contain perspectives on lived experiences as they describe characters that react to environmental cues with emotional responses.^13^ Health care professionals are exposed to patients’ accounts of their experiences every day. However, these accounts are rarely scrutinized in a systematic manner. Accordingly, grounded in narrative theory,^14,15,16^ we set out to address the question: how does type 1 diabetes fit into an emerging adult’s evolving life story?

## Methods

### Narrative Approach

The methodology we used was informed by Frank’s^16,17^ conception of narrative typology, which posits that when patients narrate illness experiences, they do so using distinct styles. By explicating the distinct styles that participants used to tell their stories, we sought to better understand how participants assigned meaning to their experiences with type 1 diabetes.^18^ Not only what is said, but how it is said, is important when using this narrative approach^12^ (ie, not only the facts contained within a statement but the words and expressions used to give meaning to the statement using analogy, context, or description, for instance). In this sense, the interviews that were performed can be considered a site for identity interpretation, in which participants spoke of who they were, who they are, and who they thought they could become.^19^

### Sampling and Recruitment

The research took place at 2 Young Adult Diabetes Clinics in Toronto, Ontario, Canada, and 1-time, in-person interviews were conducted between October 14, 2016, and May 16, 2017. Patients were eligible to participate if they were between the ages of 18 and 24 years, proficient in English, and diagnosed with type 1 diabetes for 1 year or more. Health care professionals introduced the study to eligible patients at the end of a routine clinic visit, and interested individuals met a member of the research team (B.M.), who described the study, collected contact information, and provided prospective participants with informed consent forms to review in their own time. At a later date, interested individuals were contacted (B.M.) to schedule an interview. All participants provided written informed consent. Ethics approval for the study was received from the research ethics boards of St. Michael’s Hospital and Women’s College Hospital. Given that the goal was to elicit a range of participant experiences and perceptions (but not all possible perspectives), a convenience sampling approach was used.^20^ We approached sampling broadly and were able to include participants from a wide spectrum of sociodemographic and ethnic backgrounds. We followed the Standards for Reporting Qualitative Research (SRQR) reporting guideline in the reporting of this study.

### Data Collection

Interviews were conducted by one of us (B.M.) in a private room at St. Michael’s Hospital or Women’s College Hospital. The interviewer was a master of science student at the time of conducting the interviews, with no prior relationship to any of the participants. Each interview was audio recorded and transcribed verbatim. The median (range) interview length was 58 (10-125) minutes. Interviews sought to elicit in-depth descriptions about life with diabetes from the first-person perspective.^21^ A semistructured interview guide was used to orient the dialogue between interviewer and participant^21^ (Box). The interview guide contained open-ended questions designed to help participants freely articulate past and present experiences living with diabetes, but with the freedom for participants to bring up additional topics so that the interviewer could probe further into specific experiences as necessary. In addition to the open-ended questions, participants were asked a set of demographic and clinical questions.

Box. **Interview Guide**

**Guiding Questions^a^**

1. Tell me about your experience of being diagnosed.
2. How did you learn to manage diabetes?
3. Has having diabetes changed the way that others view you?
4. Have you ever felt limited by your diabetes? Have you ever felt that diabetes has interfered with any parts of your life?
5. Have you ever felt motivated by your diabetes?
6. Tell me about your grade school experience.
7. Tell me about your high school experience.
8. Tell me about your experience attending diabetes clinic(s).
9. Have you ever traveled without your parents?
10. Have you ever attended a camp during summer time? If so, tell me about that experience.
11. What did you decide to do after high school?
12. What is a typical day like for you nowadays? Tell me about what you do.
13. How would you describe your diet nowadays?
14. Tell me about your home life/family life now.
15. Are you in a romantic relationship now?
16. Tell me about your social life nowadays.
17. Have you ever tried alcohol or recreational drugs?
18. Have you ever been involved in a religion?
19. How does diabetes affect you financially?
20. How is your life now different or the same from someone else’s your age?
21. How do you describe yourself (ie, how do you talk about yourself)?
22. Do you ever think about the future?
23. Is there anything else you would like to add?

^a^Guiding questions were used to facilitate participant storytelling and narrative analysis of participant transcripts was used to explore the meanings participants ascribed to their experiences. Each guiding question was followed by up to 8 further probes in the interview guide.

### Narrative Analysis

Transcripts were deidentified, password protected, and encrypted. Accuracy checks were performed to ensure that the transcribed text aligned with the audio recordings. A narrative analytic approach to interview transcripts was adopted.^22^ Significant features of the individual narratives, such as content, tone (language used), and form (common perspectives and discursive elements within a participant’s interview), were analyzed to interpret distinct styles of storytelling exhibited by participants.^23^ A strategy of multiple readings and constant comparison of transcripts was used throughout the analysis.^24^ Questioning and theorizing related to the evolving interpretations was iterative and sought to connect the findings to the literature.^25^ While the interviewer (B.M.) led the qualitative analysis, analytic meetings were held with all investigators throughout the study to review the analytic framework and discuss evolving interpretations. By the 22nd interview, 3 distinct types of narrative had become recognizable, and after the 33rd interview, the analytic team agreed that the narrative analysis had reached conceptual saturation, in that no further themes were identified and a justifiable narrative typology had been formulated. Following the definition of our narrative typology, a post hoc statistical analysis was performed in which participant demographic and clinical characteristics were compared according to narrative type. All participants verbally responded to a series of clinical and demographic questions asked by the interviewer during their interviews and the most recent HbA_1c_ values prior to the interview were recorded from the participant’s medical record. Participants self-reported the ethnicity with which they identified.

### Statistical Analysis

Quantitative clinical and demographic data are shown as mean (standard deviation). Continuous variables were compared by Welch analysis of variance followed by Dunnett T3 multiple-comparison test using GraphPad Prism 8 for Mac OS X (GraphPad Software) and categorical variables were compared using Fisher exact test followed by Bonferroni corrected pairwise comparison. A 2-sided *P* value less than .05 was considered statistically significant.

## Results

### Study Participants

We interviewed 33 participants (16 [49%] men and 17 [51%] women; mean [SD] age, 20.6 [1.7] years). The clinical characteristics of the study population are summarized in Table 1. The mean (SD) duration of diabetes was 12.0 (4.8) years, mean (SD) HbA_1c_ was 8.4% (1.5%), and 20 of 33 participants (61%) were using continuous subcutaneous insulin infusion (insulin pump) as their means for insulin administration.

**Table 1.** Demographic and Clinical Characteristics of the Study Population

Characteristic	No. (%)
Participants, No.	33
Men	16 (49)
Women	17 (51)
Age at interview, mean (SD), y	20.6 (1.7)
Duration of diabetes at interview, mean (SD), y	12.0 (4.8)
Hemoglobin A_1c_ at most recent visit preinterview, mean (SD), %	8.4 (1.5)
Insulin administration	
Continuous subcutaneous insulin infusion	20 (61)
Multiple daily injections	13 (39)
Ethnic identity	
White	18 (55)
Filipino	3 (9)
Middle Eastern	2 (6)
West Indian	1 (3)
Asian	1 (3)
Black	1 (3)
Latin American	1 (3)
Italian	1 (3)
South Asian	1 (3)
White and Aboriginal	1 (3)
Latin American and West Indian	1 (3)
White and Filipino	1 (3)
White and Asian	1 (3)
Sexually identify as lesbian, gay, bisexual, transgender, or queer^a^	2 (6)
In romantic relationship	13 (39)
Living arrangement	
Living with parent or caregiver	24 (73)
Living without parent or caregiver	9 (27)
Have a parent who has type 1 or type 2 diabetes	5 (15)
Have a sibling who has type 1 diabetes	4 (12)
Occupation	
University or college student	22 (67)
Full-time work	5 (15)
Part-time work	5 (15)
Unemployed	1 (3)

^a^ One participant was gay and 1 participant was bisexual.

### Narrative Typologies: Ingrained, Intrusive, and Inconspicuous Lenses

Participants’ accounts addressed common topics pertinent to emerging adult life circumstances including experiences with college and work, romantic relationships, drug and alcohol use, travel, and interactions with family members, friends, and health care professionals. Participants also spoke in depth about the practical tasks involved in daily diabetes self-management, their attitudes and the attitudes of others toward indicators of glycemic control (eg, HbA_1c_), and how living with diabetes intersected with their personal and social relationships. Over the course of analysis, we found that there were commonalities in the ways in which narratives were told that enabled us to define 3 broad narrative types, or lenses. Major themes and illustrative quotes for each lens type are summarized in Table 2.

**Table 2.** Core Narrative Themes Common to Participant Accounts Within Lens Groups

Narrative Lens	Theme	Explanation	Example Quote
Ingrained lens	Becoming a “self-doctor”	Spoke in terms of agency and expertise	“…I took it up to myself to learn as much as I can of the disease, how my body reacts to it, how I can better manage it.”
	Finding moderation	Perceived themselves as able to accommodate for diabetes within their lives and make compromises when necessary	“…So I’ve learned to be like, ‘Okay, so let me find out what I really can’t do or what I really can do with it and then just accept it,’ like, cause to try to fight something is like you’re wasting a lot of time and energy when really you can just understand it and then adapt to it.”
	Earning trust	Articulated that they had proven themselves as competent self-managers to parents and health care professionals	“They have the biggest confidence in me, in terms of, ‘Okay, he’s perfectly fine, he knows what he’s doing.’”
Intrusive lens	Playing catch-up	Described a lack of preparation for self-management as they were overwhelmed by greater autonomy	“18 is the year that, you know, you’re technically, you’re an adult, so yeah, they just kind of like pushed all those responsibilities on me and…it was overwhelming.”
	Developing moral sensitivities	Sensitive to other people’s judgement of what is right or wrong about their diabetes self-management	“When I tell them [parents] my A_1c_ they definitely take it as a score.”
Inconspicuous lens	Keeping diabetes away from the spotlight	Attempts to disregard attendant diabetes related tasks with the goal of protecting their sense of normalcy	“…I remember not really wanting to take my injections right in the cafeteria because like I was kind of self-conscious about like taking out needles and stuff.”
	Minimizing the moral context of diabetes	Expressed awareness of, but comfort with, suboptimal diabetes control or self-management (ie, they described being content managing diabetes “my way,” rather than overburden themselves with trying to manage the so-called “right way”)	“…I don't really know because my A_1c_ for the past couple of times that I've been there has been really good. But, yeah, again, I find myself really in control, at times, like obviously there have been times where I've had my own seizures and stuff, but again, they're learning experiences.”

#### An Ingrained Lens: “Diabetes Is Comfortably a Part of My Norm”

The first lens is what we came to define as an ingrained lens, conveyed by 14 of 33 participants (43%). From an ingrained perspective, participants presented themselves as trustworthy self-managers who said that diabetes and its self-management tasks caused little disturbance to their daily lives.

Participant 1: “…it’s just something that we’ve [participant and family] come to accept and it’s like, it’s just in the back the whole time.”

Not only were self-management tasks recounted as smoothly integrated into participants’ daily lives, but diabetes itself was depicted as being an integral component of their life stories.

Participant 27: “Like when you are young you feel like it’s a burden but now that I am older it’s like, it’s not really a burden it’s just part of my story. Like I’ve said, ‘Oh yeah, I’m this year, this is how old I am, I go to school, this is my background, I am a diabetic [sic].’”

In ingrained narratives, participants adopted a practical, flexible approach to self-management that helped them maintain a sense of control within their daily lives. Participants recalled childhood and adolescence as times during which they had learned the foundations of self-management, and as emerging adults they felt well equipped to manage diabetes on their own.

Participant 16: “…there’s not really a definitive point where I could say that I understood, it was more of a learning curve, like, you know, everyone learns to use the toilet, everyone learns to eat with a knife and fork, I also had to learn to take care of my diabetes…”

However, despite feeling as though they were in control, the ingrained narratives indicated that participants appeared to welcome the continued role their parents played in the management of diabetes.

Participant 27: “When I feel like I am losing my way with the diabetes they just kind of tug me back.”

Similarly, within ingrained narratives, participants portrayed health care professionals as supportive figures. As they entered the adult care setting, these participants described taking on a new, more engaged role in health care appointments in which they felt that dialogue was more mature and built around their own individual needs.

Participant 9: “…it definitely feels like a team because everyone is involved and you talk to them as though they’re, you’re on equal terms in the sense that you are all just trying to make you better.”

Overall, from the ingrained perspective, the stories of emerging adults with type 1 diabetes characterized participants as becoming diligent self-managers who learned how to work with parents and health care professionals to integrate diabetes seamlessly into their lives.

#### An Intrusive Lens: “My Diabetes Is Like a Weight, Constantly Weighing Down on Me”

The second narrative type we delineated was intrusive, which was the dominant narrative in the interviews of 12 of 33 participants (36%). These participants’ narratives articulated a tone of distress that contrasted starkly with the more positive accounts portrayed from an ingrained perspective. Participants spoke as though diabetes was a heavy burden and they voiced struggles integrating self-management tasks into their daily lives.

Participant 33: “…I will look around and be like, ‘You don’t have to do this, you don’t have to do this, why do I have to do it?’”

Participants found it difficult to stay motivated with daily self-management because they felt as though diabetes, and its attendant tasks, were intrusive both physically and psychologically.

Participant 24: “…you have to like stick stuff in yourself all the time, like always. I have like scarring on my fingers from like checking my blood glucose, which is annoying.… Sometimes I am just like, ‘I just don’t need to do this.’ In my head I am like, ‘My sugar is fine right now but I should check it.’...And then I just see that other people don’t have to do anything and it kind of makes me think…”

It seemed unfair to these participants that their peers without diabetes did not have to attend to the extra work required in diabetes self-management. Accounts about testing one’s blood glucose or injecting insulin were told dramatically and constantly remembering to test blood glucose or administer insulin was perceived as arduous. Rather than being habitual behaviors, self-management tasks intruded on the lives that participants hoped to have; these tasks were deemed unwelcome interruptions. Intrusive narratives characterized participants as feeling unprepared for self-management and overwhelmed by the expectations of autonomy as adults. There was a sense that these participants were in a catch-up state for self-management, as they recounted difficulties trying to learn from their parents.

Participant 33: “…everything I was learning was secondhand through my parents. And I think that’s where I had a lot of my downfall just because I was getting bored of the way they were doing it…”

In general, rather than gradually gaining confidence in their self-management competency, these participants tended to look back on their childhood as a time of better diabetes care and they lamented that they were no longer able to attain the level of control that they once had.

#### An Inconspicuous Lens: “I Try Not to Notice Diabetes or Make It Noticeable”

We called the third narrative type an inconspicuous lens because of the distinct minimizing language commonly used in the narratives of 7 of 33 participants (21%). At first, these narrative accounts had a superficial resemblance to those of the intrusive type, in that participants recognized that they were not managing their diabetes according to expectations, for instance, describing occasions of insulin omission or infrequent self-monitoring of blood glucose. However, unlike the intrusive lens narratives, these accounts were typified by the overarching theme of minimization rather than intrusion.

Participant 23: “I’ve always kind of wanted to have diabetes like having the minimum role in my life that it needs to…have the least amount of impact that it can have. So I have always kind of like tried to, yeah, like downplay it.”

This theme of minimization could be seen both in the efforts participants made to underplay the significance of type 1 diabetes and in the efforts participants made to limit their engagement in self-management tasks.

Participant 10: “I hid it from my friends. I still have friends that don’t know actually. I hid it from people.… I felt that when people find out they tend to like treat you differently…”

Inconspicuous narratives indicated self-consciousness in the performance of self-management tasks in front of others and that participants were prepared to forego self-management tasks altogether in an effort to preserve their sense of normalcy. Participants described using minimizing behaviors in social settings with the purpose of avoiding being under the watchful gaze of others. Moreover, at other times these types of minimizing behaviors were illustrative of avoidant coping, with the goal of circumventing unpleasant cognitions or emotions related to one’s self-management performance.

Participant 28: “…I’ve just never liked it [blood glucose testing]. There’s a couple theories going around still and I think the biggest one is that seeing my blood glucose numbers kind of depressed me a bit. So I go based off of how I’m feeling when it comes to blood glucoses and insulin now.”

In a similar vein, to avoid uncomfortable conversations with parents, participants were selective in choosing what information about their diabetes care they would share.

Participant 12: “…sometimes if I’d have like a bad A_1c_ I’d lie and tell them it was lower. Or if I, or I shouldn’t say that, I don’t like to prescribe moral value to like health stuff, but like a high A_1c_, you know, I would just say it was lower.”

In contrast to intrusive narratives, however, these participants portrayed themselves as not especially burdened by their diabetes and they seemed comfortable intentionally diverting attention away from it.

### Comparison of the Clinical Characteristics of Participants According to Narrative Lens

Having defined our 3i concepts, we compared the clinical characteristics of the study participants according to the different narrative lens types (Table 3). There were no overall differences in age, duration of diabetes, or stage of education or work between participants according to narrative lens (Table 3). In contrast, there were more women than men in the intrusive lens group (10 vs 2, respectively), and fewer participants in this group (42%) lived with a parent or guardian. Furthermore, HbA_1c_ levels were significantly higher for participants within the intrusive lens group (mean [SD] HbA_1c_, 9.3% [1.6%]) than those of participants telling ingrained narratives (mean [SD] HbA_1c_, 7.4% [0.7%]; difference, 1.9%; 95% CI, 0.8%-2.9%; *P* = .007), whereas the HbA_1c_ levels of participants conveying an inconspicuous lens fell midway between these other 2 groups (mean [SD] HbA_1c_, 8.6% [1.5%]) and did not differ significantly from them (Table 3).

**Table 3.** Comparison of the Demographic and Clinical Characteristics of Participants According to Lens Typology

Characteristic	Lens			*P* Value^a^
	Ingrained	Intrusive	Inconspicuous	
No.	14	12	7	NA
Men, No./women, No.	9/5	2/10	5/2	.02 (Post hoc test: ingrained vs intrusive, .06; ingrained vs inconspicuous, >.99; intrusive vs inconspicuous, .13)
Age at interview, mean (SD), y	20.7 (0.5)	20.8 (0.5)	19.7 (0.6)	.34
Hemoglobin A_1c_ at most recent visit preinterview, mean (SD), %	7.4 (0.7)	9.3 (1.6)	8.6 (1.5)	.008 (Post hoc test: ingrained vs intrusive, .007; ingrained vs inconspicuous, .25; intrusive vs inconspicuous, .72)
Duration of diabetes at interview, mean (SD), y	11.9 (4.8)	13.3 (4.2)	10.1 (5.8)	.44
Continuous subcutaneous insulin infusion, No./multiple daily injection, No.	11/3	7/5	2/5	.09
Living with a parent or guardian, No. (%)	12 (86)	5 (42)	7 (100)	.01 (Post hoc test: ingrained vs intrusive, .11; ingrained vs inconspicuous >.99; intrusive vs inconspicuous, .05)
University or college student, No. (%)	10 (71)	8 (67)	4 (57)	.89

Abbreviation: NA, not applicable.

^a^ Statistical comparisons by Fisher exact test followed by Bonferroni corrected pairwise comparison for categorical variables and by Welch analysis of variance followed by Dunnett T3 multiple-comparison test for continuous variables.

## Discussion

An appreciation of the psychosocial context in which health decisions are made by patients is at the cornerstone of the modern movement toward interpersonal medicine.^26^ This is especially the case when one considers decisions made in the context of a chronic disease that requires the daily attention of the patient (type 1 diabetes) at a time of life when there is tremendous change and uncertainty (emerging adulthood). Here, we present a framework that illustrates how emerging adults narrate the experience of living with type 1 diabetes. Although human behavior is more complex than our framework can reflect, recognition by health care professionals of these different viewpoints should facilitate relationship building and the tailoring of type 1 diabetes self-management for individual patients during their emerging adult years.

Early emerging adulthood is a time of great flux that can be characterized by geographic, emotional, and social mobility as individuals separate from parental care, form new relationships, and move on to higher education or full-time work.^2,27^ With the goal of informing health care professionals of the lived experience of type 1 diabetes during emerging adulthood, we analyzed narrative accounts of patients to define common typologies. This approach is distinct from previous qualitative studies of the lived experience of emerging adults with type 1 diabetes,^28,29,30,31,32,33^ which have tended to focus on the practical aspects of type 1 diabetes without delving into the manner in which accounts are articulated or the complex meanings patients ascribe to their experiences. We describe 3 narrative types articulated by emerging adults with type 1 diabetes, which we designated as the ingrained, intrusive, and inconspicuous lenses. From an ingrained lens perspective, patients view themselves as competent self-managers for whom type 1 diabetes has become comfortably integrated into their lives. From an intrusive lens perspective, patients describe type 1 diabetes as an unwelcome burden, resenting self-management tasks for interfering in their daily lives. Taking an inconspicuous lens viewpoint, emerging adults with type 1 diabetes may attempt to minimize the role that type 1 diabetes has in their lives in an effort to protect their sense of normalcy and to limit distress. These lenses differ from one another with respect to the participants’ accounts of diabetes self-management and its integration into daily life; relationships with peers, parents, and health care professionals; illness perception; and attitudes toward indicators of disease management (eg, HbA_1c_).

Although there may be overlap between lenses for any individual patient and there may be fluidity between lenses over time, we identified biomedical and sociodemographic differences between participants according to narrative typology. For instance, HbA_1c_ levels were lowest in participants who adopted ingrained narratives and highest in those with intrusive narratives. Although HbA_1c_ levels differed, we cannot determine the causality of the association between HbA_1c_ level and lens type from this data set. For example, because frequency of self-monitoring of blood glucose is associated with lower HbA_1c_ levels,^34^ participants who are more engaged in their self-management, and thus of an ingrained lens type, may be expected to have lower HbA_1c_ levels than participants who are less engaged. Conversely, higher HbA_1c_ levels have been linked to avoidant coping behavior and decreases in diabetes integration.^35^ Alternatively, residual pancreatic β-cell function is associated with lower HbA_1c_ levels^36^ and it is also possible that the narratives of individuals who have residual β-cell function may contain more positive language surrounding life with type 1 diabetes and thus be categorized as being of an ingrained lens type. Aside from HbA_1c_, we also observed that there was a preponderance of women in the intrusive lens group, which is consistent with a previous report^37^ that symptoms of diabetes distress are more common in female than male adolescents and emerging adults with type 1 diabetes. This finding is also aligned with existing research that has found gender differences in the embodiment of diabetes,^38^ along with other psychological constructs, such as coping^39^ or feelings of engulfment.^40^ However, whether actual diabetes distress differs between genders or whether the manner in which diabetes distress is articulated during interviews differs between genders cannot be resolved from the present data set.

### Limitations

Our study has several limitations. For instance, interviews were performed at a single point in time and thus reflect the perspectives of participants at the time of their interview. Participants may have presented themselves differently at another time or if interviewed by another interviewer. The findings are also dependent on context in that a different sample of emerging adults with type 1 diabetes (eg, those attending a general adult diabetes clinic or patients in a rural setting) may have identified different issues. Accordingly, further research is needed to ascertain to what extent the 3i framework described applies more generally to other patient populations or to other emerging adults living with type 1 diabetes. Also, although we observed differences in some clinical parameters between participants, according to lens typology (eg, HbA_1c_ levels), it should be borne in mind that the study design was qualitative in nature, and caution should be exercised in assigning biological meaning to any of these differences.

### Implications

Despite its limitations, the categorical conceptualization of these 3 common transitioning patient perspectives on life with type 1 diabetes does offer potentially practice-influencing insights. For example, health care professionals may consider an individual patient’s lens type in recommending particular insulin administration strategies (continuous subcutaneous insulin infusion, multiple daily injections, morning isophane insulin, or premixed insulin) or particular glucose monitoring approaches (fingerstick, real-time continuous glucose monitoring, or intermittently scanned continuous glucose monitoring). Recognition of times when emerging adults are finding self-management especially burdensome or when emerging adults are minimizing the impact of diabetes could facilitate discussions around coping strategies. Health care professionals should also recognize that there are occasions when what they may perceive as being solely a biomedical indicator (eg, HbA_1c_ results) can be assigned moralistic meaning by patients. Finally, health care professionals should be cognizant of the bidirectional relationship between diabetes self-management performance and the interactions persons with diabetes have with their friends and family members. The purpose of presenting our framework is not to seek strategies to move patients between lens types, but rather to provide a heuristic for health care practitioners to understand the different perspectives emerging adults may have on living with type 1 diabetes so that care can be individualized accordingly.

## Conclusions

Through analysis of narrative accounts, we identified 3 different overarching perspectives (or lenses) by which emerging adults with type 1 diabetes articulated their life stories. With an understanding of this 3i conceptual framework, it is hoped that health care professionals may feel better equipped to provide developmentally appropriate, individualized care to the transitioning population living with type 1 diabetes.
